# Tetra­kis(μ_2_-ferrocene­carboxyl­ato-κ^2^ *O*:*O*′)bis­[(methanol-κ*O*)copper(II)] methanol disolvate

**DOI:** 10.1107/S1600536811050185

**Published:** 2011-11-25

**Authors:** Beñat Artetxe, Pablo Vitoria, Aroa Pache, Santiago Reinoso, Juan M. Gutiérrez-Zorrilla

**Affiliations:** a Departamento de Química Inorgánica, Facultad de Ciencia y Tecnología, Universidad de País Vasco (UPV/EHU), PO Box 644, E-48080 Bilbao, Spain

## Abstract

The complex mol­ecule of the title compound, [Cu_2_Fe_4_(C_5_H_5_)_4_(C_6_H_4_O_2_)_4_(CH_3_OH)_2_]·2CH_3_OH, lies about an inversion centre and contains two centrosymetrically related Cu^II^ atoms bridged by four *O*:*O*′-bidentante ferrocene­carboxyl­ate anions, leading to a dimeric tetra­bridged unit with a paddle-wheel geometry. The Cu^II^ atom has a distorted square-pyramidal coordination environment with four O atoms from four ferrocene­carboxyl­ate ligands in basal positions and an O atom from a methanol mol­ecule in an apical position. One of the two crystallographically independent ferrocenyl groups has a staggered conformation, while the other is eclipsed. The mol­ecules are connected into a chain along the *b* axis by O—H⋯O hydrogen bonds involving coordinated and uncoordinated methanol mol­ecules and the O atom from a ferrocene­carboxyl­ate unit.

## Related literature

For related structures, see: Churchill *et al.* (1985 ▶); Cooke *et al.* (2002 ▶); Zhang *et al.* (2009 ▶).
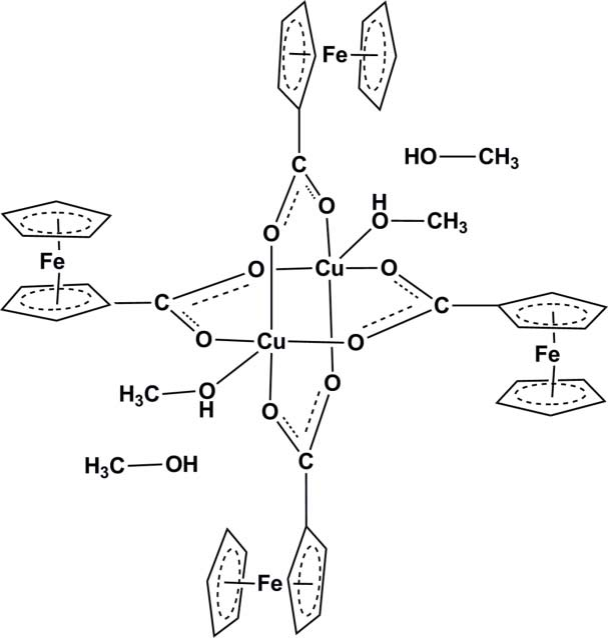


## Experimental

### 

#### Crystal data

[Cu_2_Fe_4_(C_5_H_5_)_4_(C_6_H_4_O_2_)_4_(CH_4_O)_2_]·2CH_4_O
*M* *_r_* = 1171.40
Triclinic, 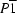

*a* = 9.5112 (8) Å
*b* = 9.5884 (9) Å
*c* = 13.2478 (14) Å
α = 72.867 (7)°
β = 79.911 (8)°
γ = 85.399 (7)°
*V* = 1136.17 (19) Å^3^
*Z* = 1
Mo *K*α radiation
μ = 2.23 mm^−1^
*T* = 100 K
0.13 × 0.10 × 0.01 mm

#### Data collection

Stoe IPDS 2T diffractometer
Absorption correction: integration (*X-RED*; Stoe & Cie, 2002 ▶) *T*_min_ = 0.768, *T*_max_ = 0.969
8197 measured reflections
3990 independent reflections
2199 reflections with *I* > 2σ(*I*)
*R*_int_ = 0.071

#### Refinement

*R*[*F* ^2^ > 2σ(*F* ^2^)] = 0.043
*wR*(*F* ^2^) = 0.080
*S* = 0.77
3990 reflections
306 parameters
2 restraints
H atoms treated by a mixture of independent and constrained refinement
Δρ_max_ = 0.38 e Å^−3^
Δρ_min_ = −0.38 e Å^−3^

### 

Data collection: *X-AREA* (Stoe & Cie, 2002 ▶); cell refinement: *X-AREA*; data reduction: *X-RED* (Stoe & Cie, 2002 ▶); program(s) used to solve structure: *SIR2004* (Burla *et al.*, 2005 ▶); program(s) used to refine structure: *SHELXL97* (Sheldrick, 2008 ▶); molecular graphics: *ORTEP-3* (Farrugia, 1997 ▶); software used to prepare material for publication: *WinGX* (Farrugia, 1999 ▶).

## Supplementary Material

Crystal structure: contains datablock(s) I, global. DOI: 10.1107/S1600536811050185/is5012sup1.cif

Structure factors: contains datablock(s) I. DOI: 10.1107/S1600536811050185/is5012Isup2.hkl

Additional supplementary materials:  crystallographic information; 3D view; checkCIF report

## Figures and Tables

**Table 1:** Hydrogen-bond geometry (Å, °)

*D*—H⋯*A*	*D*—H	H⋯*A*	*D*⋯*A*	*D*—H⋯*A*
O5—H5*O*⋯O6^i^	0.82 (6)	1.95 (6)	2.759 (6)	170 (7)
O6—H6*O*⋯O2^ii^	0.84 (4)	2.18 (5)	2.925 (6)	147 (7)

Symmetry codes: (i) 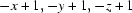; (ii) 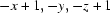.

**Fig. 1.**
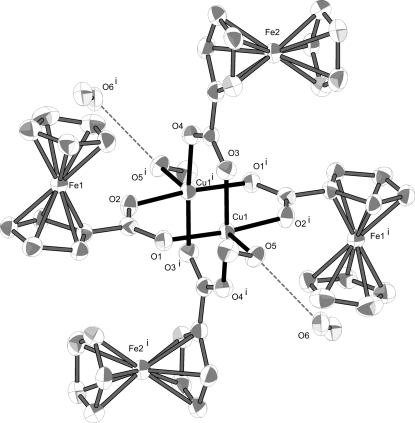
Molecular structure of (I) showing atom-labelling and 50% probability displacement ellipsoids [symmetry code: (i) -x, -y + 1, -z + 1].

**Fig. 2.**
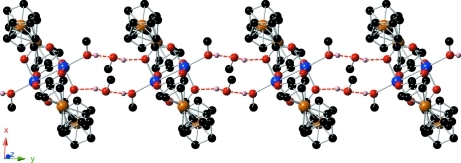
Crystal packing of (I) viewed down the c axis, showing a chain of complexes connected via O—H···O hydrogen bonds (dashed lines). H atoms not involved in hydrogen bonding have been omitted for clarity.

## supplementary crystallographic information

### Comment

The title compound, [Cu_2_{Fe(C_5_H_5_)(C_5_H_4_COO)}_4_(CH_3_OH)_2_].2CH_3_OH, was obtained in an attempted synthesis of a hybrid inorganic-metalorganic compound based on Keggin-type polyoxometalates and ferrocenecarboxylate-copper(II) complexes in methanol. Isolation of the compound was only observed in the presence of the [PW_9_O_34_]^9-^ polyanion precursor.

The title compound (Fig. 1) contains two centrosymmetrically related copper(II) centers bridged by four ferrocenecarboxylate anions (*L*) in a *O*,*O*'-bidentantate fashion, leading to a dimeric tetrabridged [Cu_2_(µ_2_-*L*)_4_] unit. Each copper(II) ion has a square-pyramidal coordination environment with four oxygen atoms from four ferrocenecarboxylate ligands and an oxygen atom from a methanol molecule in apical position. The paddle-wheel structure of the complex brings the metal centers close to each other, being the intradimer Cu···Cu^i^ distance 2.5936 (14) Å [symmetry code: (i) -*x*, -*y* + 1, -*z* + 1], shorter than 2.605 (1) Å observed in the corresponding THF complex (Churchill *et al.*, 1985). The two crystallographically independent ferrocenyl moieties have different conformations: that containing Fe1 is staggered, while that involving Fe2 is eclipsed. The crystal packing is built up by hydrogen bonding interactions involving coordinated and uncoordinated methanol molecules (Table 1). This hydrogen bonding network results in chains parallel to the *b* axis (Fig. 2). The connection among them is made by C—H···O and π–π type weak interactions.

### Experimental

CuCl_2_.2H_2_O (34 mg, 0.2 mmol), ferrocenecarboxylic acid (46 mg, 0.2 mmol) and Na_9_[PW_9_O_34_].7H_2_O (244 mg, 0.1 mmol) were refluxed for 2 h in methanol (20 ml). Dark green prismatic single crystals were obtained by slow evaporation of the resulting yellow solution after two days.

### Refinement

H atoms bonded to O atoms were located in a Fourier difference map and refined with distance restraint of O—H = 0.84 (2) Å and with *U*_iso_(H) = 1.5*U*_eq_(O). H atoms attached to C atoms were positioned geometrically (C—H = 0.95 or 0.98 Å) and refined using a riding, with *U*_iso_(H) = 1.2*U*_eq_(C) or 1.5*U*_eq_(methyl C). A rotating-group model was applied for the methyl groups.

### Crystal data

**Table:**

[Cu_2_Fe_4_(C_5_H_5_)_4_(C_6_H_4_O_2_)_4_(CH_4_O)_2_]·2CH_4_O	*Z* = 1
*M**_r_* = 1171.40	*F*(000) = 598
Triclinic, *P*1	*D*_x_ = 1.712 Mg m^−^^3^
Hall symbol: -P 1	Mo *K*α radiation, λ = 0.71073 Å
*a* = 9.5112 (8) Å	Cell parameters from 4954 reflections
*b* = 9.5884 (9) Å	θ = 2.2–25.5°
*c* = 13.2478 (14) Å	µ = 2.23 mm^−^^1^
α = 72.867 (7)°	*T* = 100 K
β = 79.911 (8)°	Plate, dark green
γ = 85.399 (7)°	0.13 × 0.10 × 0.01 mm
*V* = 1136.17 (19) Å^3^	

### Data collection

**Table:**

Stoe IPDS 2T diffractometer	3990 independent reflections
Radiation source: sealed X-ray tube, 12 x 0.4 mm long-fine focus	2199 reflections with *I* > 2σ(*I*)
plane graphite	*R*_int_ = 0.071
Detector resolution: 6.67 pixels mm^-1^	θ_max_ = 25°, θ_min_ = 2.2°
rotation method scans	*h* = −11→11
Absorption correction: integration (*X-RED*; Stoe & Cie, 2002)	*k* = −11→10
*T*_min_ = 0.768, *T*_max_ = 0.969	*l* = −15→15
8197 measured reflections	

### Refinement

**Table:**

Refinement on *F*^2^	Primary atom site location: structure-invariant direct methods
Least-squares matrix: full	Secondary atom site location: difference Fourier map
*R*[*F*^2^ > 2σ(*F*^2^)] = 0.043	Hydrogen site location: difference Fourier map
*wR*(*F*^2^) = 0.080	H atoms treated by a mixture of independent and constrained refinement
*S* = 0.77	*w* = 1/[σ^2^(*F*_o_^2^) + (0.0201*P*)^2^] where *P* = (*F*_o_^2^ + 2*F*_c_^2^)/3
3990 reflections	(Δ/σ)_max_ < 0.001
306 parameters	Δρ_max_ = 0.38 e Å^−^^3^
2 restraints	Δρ_min_ = −0.38 e Å^−^^3^

### Special details

**Table:**

Experimental. IR (cm^-1^): 1620(w), 1566(*s*), 1474(*s*), 1389(*s*), 1358(*m*), 1188(w), 1103(w), 1026(w), 1003(w), 918(w), 818(*m*), 779(*m*), 532(*m*), 478(*m*).
Geometry. All s.u.'s (except the s.u. in the dihedral angle between two l.s. planes) are estimated using the full covariance matrix. The cell s.u.'s are taken into account individually in the estimation of s.u.'s in distances, angles and torsion angles; correlations between s.u.'s in cell parameters are only used when they are defined by crystal symmetry. An approximate (isotropic) treatment of cell s.u.'s is used for estimating s.u.'s involving l.s. planes.
Refinement. Refinement of *F*^2^ against ALL reflections. The weighted *R*-factor *wR* and goodness of fit *S* are based on *F*^2^, conventional *R*-factors *R* are based on *F*, with *F* set to zero for negative *F*^2^. The threshold expression of *F*^2^ > 2σ(*F*^2^) is used only for calculating *R*-factors(gt) *etc*. and is not relevant to the choice of reflections for refinement. *R*-factors based on *F*^2^ are statistically about twice as large as those based on *F*, and *R*- factors based on ALL data will be even larger.

### Fractional atomic coordinates and isotropic or equivalent isotropic displacement parameters (Å^2^)

**Table:**

	*x*	*y*	*z*	*U*_iso_*/*U*_eq_	
Cu1	0.06133 (7)	0.61943 (7)	0.49254 (6)	0.02993 (19)	
Fe1	0.45442 (9)	0.32189 (9)	0.27785 (7)	0.0333 (2)	
Fe2	−0.18777 (9)	0.77725 (9)	0.12862 (7)	0.0356 (2)	
O1	0.2339 (4)	0.5013 (4)	0.4676 (3)	0.0356 (9)	
O2	0.1308 (4)	0.2923 (4)	0.4812 (3)	0.0340 (9)	
O3	0.0423 (4)	0.6656 (4)	0.3399 (3)	0.0342 (9)	
O4	−0.0609 (4)	0.4573 (4)	0.3524 (3)	0.0344 (9)	
C1	0.2391 (6)	0.3706 (6)	0.4635 (4)	0.0328 (13)	
C2	0.3813 (6)	0.3091 (6)	0.4320 (4)	0.0314 (13)	
C3	0.4126 (6)	0.1672 (6)	0.4197 (4)	0.0337 (13)	
H3	0.3465	0.0914	0.4363	0.040*	
C4	0.5609 (6)	0.1587 (6)	0.3779 (4)	0.0363 (14)	
H4	0.6114	0.0762	0.3619	0.044*	
C5	0.6193 (6)	0.2954 (6)	0.3648 (4)	0.0377 (14)	
H5	0.7161	0.3205	0.3376	0.045*	
C6	0.5097 (6)	0.3889 (6)	0.3988 (4)	0.0344 (14)	
H6	0.5201	0.4864	0.3993	0.041*	
C7	0.2850 (7)	0.3698 (7)	0.1955 (5)	0.0474 (16)	
H7	0.1869	0.3641	0.2264	0.057*	
C8	0.3719 (8)	0.2578 (7)	0.1675 (5)	0.0543 (18)	
H8	0.3425	0.1623	0.1755	0.065*	
C9	0.5131 (7)	0.3118 (7)	0.1244 (5)	0.0470 (16)	
H9	0.5937	0.2580	0.1004	0.056*	
C10	0.5100 (7)	0.4575 (6)	0.1243 (5)	0.0423 (15)	
H10	0.5876	0.5219	0.0987	0.051*	
C11	0.3702 (7)	0.4919 (7)	0.1694 (5)	0.0442 (16)	
H11	0.3388	0.5837	0.1802	0.053*	
C12	−0.0144 (6)	0.5822 (6)	0.3026 (5)	0.0311 (13)	
C13	−0.0278 (6)	0.6391 (6)	0.1868 (5)	0.0373 (14)	
C14	−0.1018 (6)	0.5751 (6)	0.1275 (5)	0.0399 (15)	
H14	−0.1499	0.4854	0.1546	0.048*	
C15	−0.0918 (6)	0.6670 (6)	0.0223 (5)	0.0424 (15)	
H15	−0.1323	0.6498	−0.0335	0.051*	
C16	−0.0124 (6)	0.7879 (7)	0.0132 (5)	0.0431 (16)	
H16	0.0093	0.8671	−0.0495	0.052*	
C17	0.0303 (6)	0.7727 (6)	0.1131 (5)	0.0384 (15)	
H17	0.0868	0.8385	0.1289	0.046*	
C18	−0.3181 (6)	0.8241 (6)	0.2552 (5)	0.0405 (15)	
H18	−0.3050	0.7905	0.3281	0.049*	
C19	−0.3943 (6)	0.7520 (6)	0.2055 (5)	0.0400 (15)	
H19	−0.4405	0.6619	0.2383	0.048*	
C20	−0.3906 (6)	0.8365 (6)	0.0978 (5)	0.0414 (15)	
H20	−0.4341	0.8131	0.0458	0.050*	
C21	−0.3092 (6)	0.9645 (6)	0.0808 (5)	0.0415 (15)	
H21	−0.2895	1.0408	0.0160	0.050*	
C22	−0.2635 (7)	0.9545 (7)	0.1811 (5)	0.0475 (17)	
H22	−0.2074	1.0225	0.1947	0.057*	
O5	0.1722 (4)	0.8204 (4)	0.4532 (3)	0.0361 (10)	
H5O	0.162 (7)	0.877 (6)	0.490 (4)	0.054*	
C23	0.3032 (6)	0.8342 (7)	0.3782 (5)	0.0436 (16)	
H23A	0.2984	0.7777	0.3282	0.065*	
H23B	0.3836	0.7972	0.4170	0.065*	
H23C	0.3169	0.9372	0.3383	0.065*	
O6	0.8782 (4)	0.0185 (4)	0.4019 (3)	0.0437 (10)	
H6O	0.898 (7)	−0.071 (3)	0.412 (5)	0.066*	
C24	0.9929 (6)	0.0836 (6)	0.3229 (5)	0.0448 (16)	
H24A	0.9595	0.1757	0.2767	0.067*	
H24B	1.0295	0.0174	0.2796	0.067*	
H24C	1.0693	0.1028	0.3575	0.067*	

### Atomic displacement parameters (Å^2^)

**Table:**

	*U*^11^	*U*^22^	*U*^33^	*U*^12^	*U*^13^	*U*^23^
Cu1	0.0298 (4)	0.0248 (4)	0.0363 (4)	0.0026 (3)	−0.0068 (3)	−0.0105 (3)
Fe1	0.0353 (5)	0.0302 (5)	0.0349 (5)	0.0035 (4)	−0.0067 (4)	−0.0106 (4)
Fe2	0.0375 (5)	0.0326 (5)	0.0377 (5)	0.0038 (4)	−0.0083 (4)	−0.0112 (4)
O1	0.033 (2)	0.032 (2)	0.045 (3)	0.0021 (17)	−0.0076 (18)	−0.0146 (19)
O2	0.035 (2)	0.025 (2)	0.041 (2)	0.0006 (17)	−0.0024 (18)	−0.0102 (18)
O3	0.036 (2)	0.031 (2)	0.037 (2)	−0.0037 (18)	−0.0070 (18)	−0.0093 (19)
O4	0.042 (2)	0.027 (2)	0.034 (2)	−0.0026 (18)	−0.0090 (18)	−0.0065 (18)
C1	0.035 (3)	0.031 (3)	0.031 (3)	−0.001 (3)	−0.007 (3)	−0.006 (3)
C2	0.034 (3)	0.024 (3)	0.038 (3)	0.002 (2)	−0.011 (3)	−0.011 (3)
C3	0.043 (3)	0.021 (3)	0.038 (3)	0.002 (3)	−0.006 (3)	−0.010 (3)
C4	0.035 (3)	0.037 (3)	0.037 (3)	0.005 (3)	−0.002 (3)	−0.013 (3)
C5	0.036 (3)	0.045 (4)	0.033 (3)	−0.001 (3)	−0.002 (3)	−0.014 (3)
C6	0.038 (3)	0.030 (3)	0.035 (3)	0.002 (3)	−0.006 (3)	−0.011 (3)
C7	0.042 (4)	0.060 (4)	0.043 (4)	−0.007 (3)	−0.011 (3)	−0.015 (3)
C8	0.081 (5)	0.042 (4)	0.043 (4)	−0.013 (4)	−0.020 (4)	−0.009 (3)
C9	0.050 (4)	0.055 (4)	0.035 (4)	0.008 (3)	−0.009 (3)	−0.012 (3)
C10	0.047 (4)	0.034 (4)	0.042 (4)	−0.005 (3)	−0.012 (3)	−0.002 (3)
C11	0.053 (4)	0.040 (4)	0.038 (4)	0.014 (3)	−0.011 (3)	−0.010 (3)
C12	0.026 (3)	0.032 (3)	0.036 (3)	0.003 (3)	−0.003 (3)	−0.013 (3)
C13	0.032 (3)	0.034 (3)	0.050 (4)	0.005 (3)	−0.008 (3)	−0.019 (3)
C14	0.045 (4)	0.032 (3)	0.045 (4)	0.004 (3)	−0.006 (3)	−0.017 (3)
C15	0.053 (4)	0.037 (4)	0.037 (4)	0.013 (3)	−0.014 (3)	−0.011 (3)
C16	0.044 (4)	0.041 (4)	0.039 (4)	0.009 (3)	−0.008 (3)	−0.005 (3)
C17	0.036 (3)	0.036 (3)	0.042 (4)	0.001 (3)	−0.006 (3)	−0.009 (3)
C18	0.043 (4)	0.040 (4)	0.037 (4)	0.002 (3)	−0.010 (3)	−0.009 (3)
C19	0.038 (3)	0.033 (3)	0.049 (4)	0.007 (3)	−0.005 (3)	−0.015 (3)
C20	0.044 (4)	0.045 (4)	0.040 (4)	0.004 (3)	−0.011 (3)	−0.018 (3)
C21	0.040 (3)	0.034 (3)	0.047 (4)	0.006 (3)	−0.006 (3)	−0.010 (3)
C22	0.048 (4)	0.045 (4)	0.052 (4)	0.004 (3)	0.001 (3)	−0.024 (3)
O5	0.036 (2)	0.034 (2)	0.040 (3)	−0.0005 (19)	−0.0003 (19)	−0.0169 (19)
C23	0.043 (4)	0.043 (4)	0.043 (4)	−0.003 (3)	−0.002 (3)	−0.011 (3)
O6	0.042 (2)	0.035 (2)	0.055 (3)	0.009 (2)	−0.007 (2)	−0.018 (2)
C24	0.044 (4)	0.039 (3)	0.050 (4)	0.006 (3)	−0.006 (3)	−0.012 (3)

### Geometric parameters (Å, °)

**Table:**

Cu1—O1	1.954 (4)	C6—H6	0.9500
Cu1—O4^i^	1.966 (4)	C7—C8	1.400 (9)
Cu1—O2^i^	1.970 (4)	C7—C11	1.400 (8)
Cu1—O3	1.977 (4)	C7—H7	0.9500
Cu1—O5	2.154 (4)	C8—C9	1.437 (9)
Cu1—Cu1^i^	2.5936 (14)	C8—H8	0.9500
Fe1—C2	2.009 (6)	C9—C10	1.395 (9)
Fe1—C3	2.020 (6)	C9—H9	0.9500
Fe1—C8	2.035 (7)	C10—C11	1.415 (8)
Fe1—C9	2.040 (6)	C10—H10	0.9500
Fe1—C11	2.044 (6)	C11—H11	0.9500
Fe1—C7	2.049 (6)	C12—C13	1.493 (8)
Fe1—C6	2.052 (6)	C13—C14	1.428 (8)
Fe1—C4	2.056 (6)	C13—C17	1.445 (8)
Fe1—C5	2.061 (6)	C14—C15	1.404 (8)
Fe1—C10	2.069 (6)	C14—H14	0.9500
Fe2—C13	2.038 (6)	C15—C16	1.399 (8)
Fe2—C16	2.043 (6)	C15—H15	0.9500
Fe2—C20	2.043 (6)	C16—C17	1.415 (8)
Fe2—C18	2.044 (6)	C16—H16	0.9500
Fe2—C19	2.045 (6)	C17—H17	0.9500
Fe2—C14	2.045 (6)	C18—C19	1.398 (8)
Fe2—C17	2.046 (6)	C18—C22	1.414 (8)
Fe2—C15	2.052 (6)	C18—H18	0.9500
Fe2—C21	2.053 (6)	C19—C20	1.413 (8)
Fe2—C22	2.053 (6)	C19—H19	0.9500
O1—C1	1.267 (6)	C20—C21	1.444 (8)
O2—C1	1.271 (6)	C20—H20	0.9500
O2—Cu1^i^	1.970 (4)	C21—C22	1.444 (9)
O3—C12	1.253 (6)	C21—H21	0.9500
O4—C12	1.258 (6)	C22—H22	0.9500
O4—Cu1^i^	1.966 (4)	O5—C23	1.440 (7)
C1—C2	1.474 (7)	O5—H5O	0.82 (2)
C2—C3	1.419 (7)	C23—H23A	0.9800
C2—C6	1.426 (7)	C23—H23B	0.9800
C3—C4	1.428 (7)	C23—H23C	0.9800
C3—H3	0.9500	O6—C24	1.414 (7)
C4—C5	1.416 (8)	O6—H6O	0.84 (2)
C4—H4	0.9500	C24—H24A	0.9800
C5—C6	1.420 (7)	C24—H24B	0.9800
C5—H5	0.9500	C24—H24C	0.9800
			
O1—Cu1—O4^i^	89.58 (16)	C3—C4—Fe1	68.1 (3)
O1—Cu1—O2^i^	169.83 (16)	C5—C4—H4	126.2
O4^i^—Cu1—O2^i^	90.21 (16)	C3—C4—H4	126.2
O1—Cu1—O3	89.92 (16)	Fe1—C4—H4	127.2
O4^i^—Cu1—O3	169.82 (16)	C4—C5—C6	108.9 (5)
O2^i^—Cu1—O3	88.50 (15)	C4—C5—Fe1	69.7 (3)
O1—Cu1—O5	94.70 (15)	C6—C5—Fe1	69.5 (3)
O4^i^—Cu1—O5	102.23 (16)	C4—C5—H5	125.5
O2^i^—Cu1—O5	95.28 (15)	C6—C5—H5	125.5
O3—Cu1—O5	87.94 (16)	Fe1—C5—H5	126.9
O1—Cu1—Cu1^i^	82.06 (11)	C5—C6—C2	107.2 (5)
O4^i^—Cu1—Cu1^i^	86.42 (11)	C5—C6—Fe1	70.1 (3)
O2^i^—Cu1—Cu1^i^	87.77 (11)	C2—C6—Fe1	67.8 (3)
O3—Cu1—Cu1^i^	83.44 (11)	C5—C6—H6	126.4
O5—Cu1—Cu1^i^	170.78 (12)	C2—C6—H6	126.4
C2—Fe1—C3	41.3 (2)	Fe1—C6—H6	127.2
C2—Fe1—C8	132.1 (3)	C8—C7—C11	107.0 (6)
C3—Fe1—C8	108.0 (2)	C8—C7—Fe1	69.4 (4)
C2—Fe1—C9	172.6 (3)	C11—C7—Fe1	69.8 (4)
C3—Fe1—C9	132.8 (2)	C8—C7—H7	126.5
C8—Fe1—C9	41.3 (3)	C11—C7—H7	126.5
C2—Fe1—C11	115.1 (2)	Fe1—C7—H7	125.8
C3—Fe1—C11	145.2 (2)	C7—C8—C9	108.5 (6)
C8—Fe1—C11	67.0 (3)	C7—C8—Fe1	70.5 (4)
C9—Fe1—C11	67.4 (3)	C9—C8—Fe1	69.6 (4)
C2—Fe1—C7	108.4 (2)	C7—C8—H8	125.7
C3—Fe1—C7	113.3 (2)	C9—C8—H8	125.7
C8—Fe1—C7	40.1 (3)	Fe1—C8—H8	125.8
C9—Fe1—C7	68.5 (3)	C10—C9—C8	107.5 (6)
C11—Fe1—C7	40.0 (2)	C10—C9—Fe1	71.3 (4)
C2—Fe1—C6	41.1 (2)	C8—C9—Fe1	69.1 (4)
C3—Fe1—C6	69.1 (2)	C10—C9—H9	126.3
C8—Fe1—C6	172.3 (3)	C8—C9—H9	126.3
C9—Fe1—C6	145.8 (2)	Fe1—C9—H9	124.9
C11—Fe1—C6	111.1 (2)	C9—C10—C11	107.4 (6)
C7—Fe1—C6	133.7 (2)	C9—C10—Fe1	69.0 (3)
C2—Fe1—C4	68.9 (2)	C11—C10—Fe1	68.9 (3)
C3—Fe1—C4	41.0 (2)	C9—C10—H10	126.3
C8—Fe1—C4	114.5 (3)	C11—C10—H10	126.3
C9—Fe1—C4	109.3 (2)	Fe1—C10—H10	127.3
C11—Fe1—C4	173.7 (2)	C7—C11—C10	109.6 (6)
C7—Fe1—C4	144.8 (2)	C7—C11—Fe1	70.2 (4)
C6—Fe1—C4	68.4 (2)	C10—C11—Fe1	70.9 (3)
C2—Fe1—C5	68.5 (2)	C7—C11—H11	125.2
C3—Fe1—C5	68.4 (2)	C10—C11—H11	125.2
C8—Fe1—C5	146.1 (3)	Fe1—C11—H11	125.3
C9—Fe1—C5	115.2 (2)	O3—C12—O4	126.8 (5)
C11—Fe1—C5	135.4 (3)	O3—C12—C13	115.7 (5)
C7—Fe1—C5	173.6 (3)	O4—C12—C13	117.5 (5)
C6—Fe1—C5	40.4 (2)	C14—C13—C17	106.4 (5)
C4—Fe1—C5	40.2 (2)	C14—C13—C12	127.4 (5)
C2—Fe1—C10	146.4 (2)	C17—C13—C12	126.2 (5)
C3—Fe1—C10	172.2 (2)	C14—C13—Fe2	69.8 (3)
C8—Fe1—C10	67.6 (3)	C17—C13—Fe2	69.6 (3)
C9—Fe1—C10	39.7 (2)	C12—C13—Fe2	124.4 (4)
C11—Fe1—C10	40.2 (2)	C15—C14—C13	108.7 (5)
C7—Fe1—C10	67.9 (3)	C15—C14—Fe2	70.2 (3)
C6—Fe1—C10	116.2 (2)	C13—C14—Fe2	69.2 (3)
C4—Fe1—C10	133.9 (2)	C15—C14—H14	125.7
C5—Fe1—C10	111.3 (2)	C13—C14—H14	125.7
C13—Fe2—C16	68.9 (2)	Fe2—C14—H14	126.5
C13—Fe2—C20	156.2 (2)	C16—C15—C14	108.7 (6)
C16—Fe2—C20	124.2 (2)	C16—C15—Fe2	69.7 (3)
C13—Fe2—C18	107.8 (2)	C14—C15—Fe2	69.7 (3)
C16—Fe2—C18	157.8 (3)	C16—C15—H15	125.7
C20—Fe2—C18	67.7 (2)	C14—C15—H15	125.7
C13—Fe2—C19	121.1 (2)	Fe2—C15—H15	126.5
C16—Fe2—C19	160.7 (3)	C15—C16—C17	108.6 (5)
C20—Fe2—C19	40.4 (2)	C15—C16—Fe2	70.4 (3)
C18—Fe2—C19	40.0 (2)	C17—C16—Fe2	69.9 (3)
C13—Fe2—C14	40.9 (2)	C15—C16—H16	125.7
C16—Fe2—C14	67.7 (2)	C17—C16—H16	125.7
C20—Fe2—C14	121.1 (2)	Fe2—C16—H16	125.6
C18—Fe2—C14	125.1 (2)	C16—C17—C13	107.6 (6)
C19—Fe2—C14	108.0 (2)	C16—C17—Fe2	69.6 (4)
C13—Fe2—C17	41.4 (2)	C13—C17—Fe2	69.0 (3)
C16—Fe2—C17	40.5 (2)	C16—C17—H17	126.2
C20—Fe2—C17	160.8 (2)	C13—C17—H17	126.2
C18—Fe2—C17	122.5 (2)	Fe2—C17—H17	126.8
C19—Fe2—C17	157.4 (2)	C19—C18—C22	109.9 (6)
C14—Fe2—C17	68.4 (2)	C19—C18—Fe2	70.1 (3)
C13—Fe2—C15	68.5 (2)	C22—C18—Fe2	70.1 (3)
C16—Fe2—C15	40.0 (2)	C19—C18—H18	125.1
C20—Fe2—C15	107.8 (2)	C22—C18—H18	125.1
C18—Fe2—C15	161.1 (2)	Fe2—C18—H18	126.3
C19—Fe2—C15	124.8 (3)	C18—C19—C20	108.2 (5)
C14—Fe2—C15	40.1 (2)	C18—C19—Fe2	70.0 (3)
C17—Fe2—C15	67.8 (2)	C20—C19—Fe2	69.7 (3)
C13—Fe2—C21	160.9 (2)	C18—C19—H19	125.9
C16—Fe2—C21	107.3 (2)	C20—C19—H19	125.9
C20—Fe2—C21	41.3 (2)	Fe2—C19—H19	126.0
C18—Fe2—C21	68.2 (2)	C19—C20—C21	108.1 (5)
C19—Fe2—C21	68.7 (2)	C19—C20—Fe2	69.9 (3)
C14—Fe2—C21	156.5 (2)	C21—C20—Fe2	69.7 (3)
C17—Fe2—C21	123.7 (2)	C19—C20—H20	126.0
C15—Fe2—C21	121.4 (2)	C21—C20—H20	126.0
C13—Fe2—C22	123.8 (3)	Fe2—C20—H20	126.0
C16—Fe2—C22	121.9 (2)	C22—C21—C20	106.9 (5)
C20—Fe2—C22	69.0 (3)	C22—C21—Fe2	69.4 (3)
C18—Fe2—C22	40.4 (2)	C20—C21—Fe2	69.0 (3)
C19—Fe2—C22	68.3 (2)	C22—C21—H21	126.6
C14—Fe2—C22	161.1 (3)	C20—C21—H21	126.6
C17—Fe2—C22	107.4 (3)	Fe2—C21—H21	126.6
C15—Fe2—C22	157.2 (2)	C18—C22—C21	107.0 (6)
C21—Fe2—C22	41.2 (2)	C18—C22—Fe2	69.5 (4)
C1—O1—Cu1	126.4 (4)	C21—C22—Fe2	69.4 (3)
C1—O2—Cu1^i^	118.9 (3)	C18—C22—H22	126.5
C12—O3—Cu1	123.1 (3)	C21—C22—H22	126.5
C12—O4—Cu1^i^	120.1 (4)	Fe2—C22—H22	126.2
O1—C1—O2	124.7 (5)	C23—O5—Cu1	117.1 (3)
O1—C1—C2	116.6 (5)	C23—O5—H5O	115 (5)
O2—C1—C2	118.7 (5)	Cu1—O5—H5O	125 (4)
C3—C2—C6	108.4 (5)	O5—C23—H23A	109.5
C3—C2—C1	126.6 (5)	O5—C23—H23B	109.5
C6—C2—C1	124.8 (5)	H23A—C23—H23B	109.5
C3—C2—Fe1	69.8 (3)	O5—C23—H23C	109.5
C6—C2—Fe1	71.1 (3)	H23A—C23—H23C	109.5
C1—C2—Fe1	120.6 (4)	H23B—C23—H23C	109.5
C2—C3—C4	107.9 (5)	C24—O6—H6O	102 (5)
C2—C3—Fe1	69.0 (3)	O6—C24—H24A	109.5
C4—C3—Fe1	70.9 (3)	O6—C24—H24B	109.5
C2—C3—H3	126.1	H24A—C24—H24B	109.5
C4—C3—H3	126.1	O6—C24—H24C	109.5
Fe1—C3—H3	125.7	H24A—C24—H24C	109.5
C5—C4—C3	107.6 (5)	H24B—C24—H24C	109.5
C5—C4—Fe1	70.1 (3)		
			
O4^i^—Cu1—O1—C1	88.1 (4)	C6—Fe1—C11—C10	106.0 (4)
O2^i^—Cu1—O1—C1	−0.8 (13)	C4—Fe1—C11—C10	22 (2)
O3—Cu1—O1—C1	−81.8 (4)	C5—Fe1—C11—C10	66.5 (5)
O5—Cu1—O1—C1	−169.7 (4)	Cu1—O3—C12—O4	−3.5 (7)
Cu1^i^—Cu1—O1—C1	1.6 (4)	Cu1—O3—C12—C13	176.3 (3)
O1—Cu1—O3—C12	82.8 (4)	Cu1^i^—O4—C12—O3	4.6 (7)
O4^i^—Cu1—O3—C12	−4.4 (11)	Cu1^i^—O4—C12—C13	−175.2 (3)
O2^i^—Cu1—O3—C12	−87.1 (4)	O3—C12—C13—C14	−172.3 (5)
O5—Cu1—O3—C12	177.5 (4)	O4—C12—C13—C14	7.6 (8)
Cu1^i^—Cu1—O3—C12	0.8 (4)	O3—C12—C13—C17	6.3 (8)
Cu1—O1—C1—O2	−4.2 (8)	O4—C12—C13—C17	−173.8 (5)
Cu1—O1—C1—C2	173.5 (4)	O3—C12—C13—Fe2	−82.4 (6)
Cu1^i^—O2—C1—O1	4.5 (7)	O4—C12—C13—Fe2	97.5 (5)
Cu1^i^—O2—C1—C2	−173.2 (4)	C16—Fe2—C13—C14	−79.9 (4)
O1—C1—C2—C3	−178.5 (5)	C20—Fe2—C13—C14	48.5 (7)
O2—C1—C2—C3	−0.6 (9)	C18—Fe2—C13—C14	123.4 (3)
O1—C1—C2—C6	−4.9 (9)	C19—Fe2—C13—C14	81.7 (4)
O2—C1—C2—C6	173.0 (5)	C17—Fe2—C13—C14	−117.2 (5)
O1—C1—C2—Fe1	−92.1 (6)	C15—Fe2—C13—C14	−36.8 (3)
O2—C1—C2—Fe1	85.8 (6)	C21—Fe2—C13—C14	−161.8 (7)
C8—Fe1—C2—C3	66.4 (5)	C22—Fe2—C13—C14	165.1 (3)
C9—Fe1—C2—C3	40 (2)	C16—Fe2—C13—C17	37.4 (4)
C11—Fe1—C2—C3	147.3 (3)	C20—Fe2—C13—C17	165.7 (5)
C7—Fe1—C2—C3	104.6 (4)	C18—Fe2—C13—C17	−119.3 (4)
C6—Fe1—C2—C3	−118.8 (5)	C19—Fe2—C13—C17	−161.1 (3)
C4—Fe1—C2—C3	−38.0 (3)	C14—Fe2—C13—C17	117.2 (5)
C5—Fe1—C2—C3	−81.4 (4)	C15—Fe2—C13—C17	80.4 (4)
C10—Fe1—C2—C3	−177.8 (4)	C21—Fe2—C13—C17	−44.6 (9)
C3—Fe1—C2—C6	118.8 (5)	C22—Fe2—C13—C17	−77.7 (4)
C8—Fe1—C2—C6	−174.7 (4)	C16—Fe2—C13—C12	158.0 (6)
C9—Fe1—C2—C6	158.7 (18)	C20—Fe2—C13—C12	−73.7 (8)
C11—Fe1—C2—C6	−93.9 (4)	C18—Fe2—C13—C12	1.3 (6)
C7—Fe1—C2—C6	−136.6 (3)	C19—Fe2—C13—C12	−40.4 (6)
C4—Fe1—C2—C6	80.8 (3)	C14—Fe2—C13—C12	−122.1 (6)
C5—Fe1—C2—C6	37.5 (3)	C17—Fe2—C13—C12	120.6 (6)
C10—Fe1—C2—C6	−59.0 (5)	C15—Fe2—C13—C12	−159.0 (6)
C3—Fe1—C2—C1	−121.3 (6)	C21—Fe2—C13—C12	76.0 (9)
C8—Fe1—C2—C1	−54.9 (6)	C22—Fe2—C13—C12	42.9 (6)
C9—Fe1—C2—C1	−81 (2)	C17—C13—C14—C15	−1.0 (6)
C11—Fe1—C2—C1	26.0 (5)	C12—C13—C14—C15	177.8 (5)
C7—Fe1—C2—C1	−16.7 (5)	Fe2—C13—C14—C15	59.3 (4)
C6—Fe1—C2—C1	119.9 (6)	C17—C13—C14—Fe2	−60.3 (4)
C4—Fe1—C2—C1	−159.3 (5)	C12—C13—C14—Fe2	118.5 (5)
C5—Fe1—C2—C1	157.3 (5)	C13—Fe2—C14—C15	−120.0 (5)
C10—Fe1—C2—C1	60.9 (6)	C16—Fe2—C14—C15	−36.9 (4)
C6—C2—C3—C4	−0.4 (6)	C20—Fe2—C14—C15	80.6 (4)
C1—C2—C3—C4	174.1 (5)	C18—Fe2—C14—C15	163.8 (4)
Fe1—C2—C3—C4	60.5 (4)	C19—Fe2—C14—C15	123.0 (4)
C6—C2—C3—Fe1	−60.9 (4)	C17—Fe2—C14—C15	−80.7 (4)
C1—C2—C3—Fe1	113.6 (6)	C21—Fe2—C14—C15	45.2 (7)
C8—Fe1—C3—C2	−134.4 (4)	C22—Fe2—C14—C15	−161.5 (7)
C9—Fe1—C3—C2	−173.5 (4)	C16—Fe2—C14—C13	83.1 (4)
C11—Fe1—C3—C2	−59.1 (5)	C20—Fe2—C14—C13	−159.4 (3)
C7—Fe1—C3—C2	−91.8 (4)	C18—Fe2—C14—C13	−76.2 (4)
C6—Fe1—C3—C2	38.1 (3)	C19—Fe2—C14—C13	−117.0 (4)
C4—Fe1—C3—C2	118.8 (5)	C17—Fe2—C14—C13	39.3 (3)
C5—Fe1—C3—C2	81.6 (4)	C15—Fe2—C14—C13	120.0 (5)
C10—Fe1—C3—C2	171.1 (18)	C21—Fe2—C14—C13	165.2 (5)
C2—Fe1—C3—C4	−118.8 (5)	C22—Fe2—C14—C13	−41.5 (9)
C8—Fe1—C3—C4	106.8 (4)	C13—C14—C15—C16	0.2 (6)
C9—Fe1—C3—C4	67.7 (5)	Fe2—C14—C15—C16	58.9 (4)
C11—Fe1—C3—C4	−177.8 (4)	C13—C14—C15—Fe2	−58.7 (4)
C7—Fe1—C3—C4	149.5 (3)	C13—Fe2—C15—C16	−82.5 (4)
C6—Fe1—C3—C4	−80.7 (3)	C20—Fe2—C15—C16	122.5 (4)
C5—Fe1—C3—C4	−37.2 (3)	C18—Fe2—C15—C16	−164.6 (7)
C10—Fe1—C3—C4	52 (2)	C19—Fe2—C15—C16	163.9 (3)
C2—C3—C4—C5	−0.1 (7)	C14—Fe2—C15—C16	−120.1 (5)
Fe1—C3—C4—C5	59.1 (4)	C17—Fe2—C15—C16	−37.7 (3)
C2—C3—C4—Fe1	−59.3 (4)	C21—Fe2—C15—C16	79.2 (4)
C2—Fe1—C4—C5	−81.2 (3)	C22—Fe2—C15—C16	44.5 (8)
C3—Fe1—C4—C5	−119.5 (4)	C13—Fe2—C15—C14	37.6 (3)
C8—Fe1—C4—C5	150.9 (3)	C16—Fe2—C15—C14	120.1 (5)
C9—Fe1—C4—C5	106.5 (4)	C20—Fe2—C15—C14	−117.5 (4)
C11—Fe1—C4—C5	49 (2)	C18—Fe2—C15—C14	−44.5 (9)
C7—Fe1—C4—C5	−173.6 (4)	C19—Fe2—C15—C14	−76.1 (4)
C6—Fe1—C4—C5	−36.9 (3)	C17—Fe2—C15—C14	82.4 (4)
C10—Fe1—C4—C5	69.1 (4)	C21—Fe2—C15—C14	−160.7 (3)
C2—Fe1—C4—C3	38.3 (3)	C22—Fe2—C15—C14	164.6 (6)
C8—Fe1—C4—C3	−89.7 (4)	C14—C15—C16—C17	0.7 (7)
C9—Fe1—C4—C3	−134.0 (3)	Fe2—C15—C16—C17	59.6 (4)
C11—Fe1—C4—C3	169 (2)	C14—C15—C16—Fe2	−59.0 (4)
C7—Fe1—C4—C3	−54.1 (5)	C13—Fe2—C16—C15	81.2 (4)
C6—Fe1—C4—C3	82.5 (3)	C20—Fe2—C16—C15	−76.3 (4)
C5—Fe1—C4—C3	119.5 (4)	C18—Fe2—C16—C15	166.8 (6)
C10—Fe1—C4—C3	−171.4 (4)	C19—Fe2—C16—C15	−43.7 (9)
C3—C4—C5—C6	0.6 (7)	C14—Fe2—C16—C15	37.0 (4)
Fe1—C4—C5—C6	58.5 (4)	C17—Fe2—C16—C15	119.4 (5)
C3—C4—C5—Fe1	−57.9 (4)	C21—Fe2—C16—C15	−118.6 (4)
C2—Fe1—C5—C4	82.4 (3)	C22—Fe2—C16—C15	−161.3 (4)
C3—Fe1—C5—C4	37.9 (3)	C13—Fe2—C16—C17	−38.2 (3)
C8—Fe1—C5—C4	−52.5 (6)	C20—Fe2—C16—C17	164.3 (3)
C9—Fe1—C5—C4	−90.6 (4)	C18—Fe2—C16—C17	47.4 (8)
C11—Fe1—C5—C4	−173.2 (3)	C19—Fe2—C16—C17	−163.2 (6)
C7—Fe1—C5—C4	145 (2)	C14—Fe2—C16—C17	−82.4 (4)
C6—Fe1—C5—C4	120.5 (5)	C15—Fe2—C16—C17	−119.4 (5)
C10—Fe1—C5—C4	−133.7 (3)	C21—Fe2—C16—C17	122.0 (4)
C2—Fe1—C5—C6	−38.1 (3)	C22—Fe2—C16—C17	79.3 (4)
C3—Fe1—C5—C6	−82.6 (3)	C15—C16—C17—C13	−1.3 (6)
C8—Fe1—C5—C6	−173.0 (4)	Fe2—C16—C17—C13	58.7 (4)
C9—Fe1—C5—C6	148.9 (3)	C15—C16—C17—Fe2	−59.9 (4)
C11—Fe1—C5—C6	66.3 (4)	C14—C13—C17—C16	1.4 (6)
C7—Fe1—C5—C6	24 (2)	C12—C13—C17—C16	−177.5 (5)
C4—Fe1—C5—C6	−120.5 (5)	Fe2—C13—C17—C16	−59.1 (4)
C10—Fe1—C5—C6	105.8 (4)	C14—C13—C17—Fe2	60.5 (4)
C4—C5—C6—C2	−0.8 (7)	C12—C13—C17—Fe2	−118.4 (5)
Fe1—C5—C6—C2	57.9 (4)	C13—Fe2—C17—C16	119.3 (5)
C4—C5—C6—Fe1	−58.7 (4)	C20—Fe2—C17—C16	−43.0 (9)
C3—C2—C6—C5	0.7 (6)	C18—Fe2—C17—C16	−160.7 (3)
C1—C2—C6—C5	−173.9 (5)	C19—Fe2—C17—C16	165.6 (6)
Fe1—C2—C6—C5	−59.3 (4)	C14—Fe2—C17—C16	80.5 (4)
C3—C2—C6—Fe1	60.0 (4)	C15—Fe2—C17—C16	37.2 (3)
C1—C2—C6—Fe1	−114.6 (6)	C21—Fe2—C17—C16	−76.7 (4)
C2—Fe1—C6—C5	119.1 (5)	C22—Fe2—C17—C16	−119.1 (4)
C3—Fe1—C6—C5	80.9 (4)	C16—Fe2—C17—C13	−119.3 (5)
C8—Fe1—C6—C5	149.7 (18)	C20—Fe2—C17—C13	−162.3 (7)
C9—Fe1—C6—C5	−56.1 (6)	C18—Fe2—C17—C13	80.0 (4)
C11—Fe1—C6—C5	−136.5 (3)	C19—Fe2—C17—C13	46.3 (8)
C7—Fe1—C6—C5	−176.4 (4)	C14—Fe2—C17—C13	−38.8 (3)
C4—Fe1—C6—C5	36.8 (3)	C15—Fe2—C17—C13	−82.1 (4)
C10—Fe1—C6—C5	−92.7 (4)	C21—Fe2—C17—C13	164.0 (3)
C3—Fe1—C6—C2	−38.2 (3)	C22—Fe2—C17—C13	121.6 (4)
C8—Fe1—C6—C2	31 (2)	C13—Fe2—C18—C19	−117.4 (4)
C9—Fe1—C6—C2	−175.2 (4)	C16—Fe2—C18—C19	164.8 (6)
C11—Fe1—C6—C2	104.4 (3)	C20—Fe2—C18—C19	37.7 (3)
C7—Fe1—C6—C2	64.5 (4)	C14—Fe2—C18—C19	−75.5 (4)
C4—Fe1—C6—C2	−82.4 (3)	C17—Fe2—C18—C19	−160.6 (3)
C5—Fe1—C6—C2	−119.1 (5)	C15—Fe2—C18—C19	−42.0 (9)
C10—Fe1—C6—C2	148.1 (3)	C21—Fe2—C18—C19	82.4 (4)
C2—Fe1—C7—C8	−134.6 (4)	C22—Fe2—C18—C19	121.1 (5)
C3—Fe1—C7—C8	−90.6 (4)	C13—Fe2—C18—C22	121.5 (4)
C9—Fe1—C7—C8	38.1 (4)	C16—Fe2—C18—C22	43.8 (8)
C11—Fe1—C7—C8	118.1 (5)	C20—Fe2—C18—C22	−83.3 (4)
C6—Fe1—C7—C8	−173.3 (4)	C19—Fe2—C18—C22	−121.1 (5)
C4—Fe1—C7—C8	−55.3 (6)	C14—Fe2—C18—C22	163.5 (4)
C5—Fe1—C7—C8	165 (2)	C17—Fe2—C18—C22	78.3 (4)
C10—Fe1—C7—C8	81.0 (4)	C15—Fe2—C18—C22	−163.0 (7)
C2—Fe1—C7—C11	107.3 (4)	C21—Fe2—C18—C22	−38.6 (4)
C3—Fe1—C7—C11	151.3 (3)	C22—C18—C19—C20	−0.5 (6)
C8—Fe1—C7—C11	−118.1 (5)	Fe2—C18—C19—C20	−59.4 (4)
C9—Fe1—C7—C11	−79.9 (4)	C22—C18—C19—Fe2	59.0 (4)
C6—Fe1—C7—C11	68.6 (5)	C13—Fe2—C19—C18	80.6 (4)
C4—Fe1—C7—C11	−173.3 (4)	C16—Fe2—C19—C18	−162.6 (7)
C5—Fe1—C7—C11	47 (2)	C20—Fe2—C19—C18	−119.3 (5)
C10—Fe1—C7—C11	−37.1 (4)	C14—Fe2—C19—C18	123.6 (4)
C11—C7—C8—C9	0.6 (7)	C17—Fe2—C19—C18	46.7 (7)
Fe1—C7—C8—C9	−59.4 (5)	C15—Fe2—C19—C18	164.7 (3)
C11—C7—C8—Fe1	60.0 (4)	C21—Fe2—C19—C18	−81.1 (4)
C2—Fe1—C8—C7	65.6 (5)	C22—Fe2—C19—C18	−36.7 (4)
C3—Fe1—C8—C7	105.1 (4)	C13—Fe2—C19—C20	−160.1 (3)
C9—Fe1—C8—C7	−119.4 (5)	C16—Fe2—C19—C20	−43.3 (8)
C11—Fe1—C8—C7	−38.1 (4)	C18—Fe2—C19—C20	119.3 (5)
C6—Fe1—C8—C7	39 (2)	C14—Fe2—C19—C20	−117.1 (4)
C4—Fe1—C8—C7	148.7 (4)	C17—Fe2—C19—C20	166.0 (6)
C5—Fe1—C8—C7	−177.0 (4)	C15—Fe2—C19—C20	−76.0 (4)
C10—Fe1—C8—C7	−81.8 (4)	C21—Fe2—C19—C20	38.2 (3)
C2—Fe1—C8—C9	−175.0 (4)	C22—Fe2—C19—C20	82.6 (4)
C3—Fe1—C8—C9	−135.5 (4)	C18—C19—C20—C21	0.2 (6)
C11—Fe1—C8—C9	81.4 (4)	Fe2—C19—C20—C21	−59.5 (4)
C7—Fe1—C8—C9	119.4 (5)	C18—C19—C20—Fe2	59.6 (4)
C6—Fe1—C8—C9	158.3 (17)	C13—Fe2—C20—C19	46.4 (7)
C4—Fe1—C8—C9	−91.9 (4)	C16—Fe2—C20—C19	164.1 (3)
C5—Fe1—C8—C9	−57.6 (6)	C18—Fe2—C20—C19	−37.3 (3)
C10—Fe1—C8—C9	37.6 (4)	C14—Fe2—C20—C19	81.3 (4)
C7—C8—C9—C10	−1.4 (7)	C17—Fe2—C20—C19	−163.5 (7)
Fe1—C8—C9—C10	−61.3 (4)	C15—Fe2—C20—C19	123.1 (4)
C7—C8—C9—Fe1	60.0 (5)	C21—Fe2—C20—C19	−119.2 (5)
C2—Fe1—C9—C10	148.0 (18)	C22—Fe2—C20—C19	−80.8 (4)
C3—Fe1—C9—C10	−176.7 (4)	C13—Fe2—C20—C21	165.6 (5)
C8—Fe1—C9—C10	117.9 (6)	C16—Fe2—C20—C21	−76.7 (4)
C11—Fe1—C9—C10	37.5 (4)	C18—Fe2—C20—C21	81.9 (4)
C7—Fe1—C9—C10	80.8 (4)	C19—Fe2—C20—C21	119.2 (5)
C6—Fe1—C9—C10	−57.1 (6)	C14—Fe2—C20—C21	−159.5 (3)
C4—Fe1—C9—C10	−136.7 (4)	C17—Fe2—C20—C21	−44.3 (9)
C5—Fe1—C9—C10	−93.5 (4)	C15—Fe2—C20—C21	−117.7 (4)
C2—Fe1—C9—C8	30 (2)	C22—Fe2—C20—C21	38.3 (3)
C3—Fe1—C9—C8	65.4 (5)	C19—C20—C21—C22	0.2 (6)
C11—Fe1—C9—C8	−80.4 (4)	Fe2—C20—C21—C22	−59.3 (4)
C7—Fe1—C9—C8	−37.1 (4)	C19—C20—C21—Fe2	59.6 (4)
C6—Fe1—C9—C8	−174.9 (4)	C13—Fe2—C21—C22	−43.6 (9)
C4—Fe1—C9—C8	105.4 (4)	C16—Fe2—C21—C22	−119.0 (4)
C5—Fe1—C9—C8	148.6 (4)	C20—Fe2—C21—C22	118.4 (5)
C10—Fe1—C9—C8	−117.9 (6)	C18—Fe2—C21—C22	37.9 (3)
C8—C9—C10—C11	1.6 (7)	C19—Fe2—C21—C22	81.0 (4)
Fe1—C9—C10—C11	−58.4 (4)	C14—Fe2—C21—C22	167.3 (5)
C8—C9—C10—Fe1	60.0 (4)	C17—Fe2—C21—C22	−77.6 (4)
C2—Fe1—C10—C9	−172.9 (4)	C15—Fe2—C21—C22	−160.4 (4)
C3—Fe1—C10—C9	18 (2)	C13—Fe2—C21—C20	−162.1 (7)
C8—Fe1—C10—C9	−39.1 (4)	C16—Fe2—C21—C20	122.5 (4)
C11—Fe1—C10—C9	−119.5 (6)	C18—Fe2—C21—C20	−80.5 (4)
C7—Fe1—C10—C9	−82.6 (4)	C19—Fe2—C21—C20	−37.4 (3)
C6—Fe1—C10—C9	148.3 (4)	C14—Fe2—C21—C20	48.8 (7)
C4—Fe1—C10—C9	63.9 (5)	C17—Fe2—C21—C20	164.0 (3)
C5—Fe1—C10—C9	104.3 (4)	C15—Fe2—C21—C20	81.2 (4)
C2—Fe1—C10—C11	−53.4 (6)	C22—Fe2—C21—C20	−118.4 (5)
C3—Fe1—C10—C11	137.3 (18)	C19—C18—C22—C21	0.6 (6)
C8—Fe1—C10—C11	80.3 (4)	Fe2—C18—C22—C21	59.5 (4)
C9—Fe1—C10—C11	119.5 (6)	C19—C18—C22—Fe2	−58.9 (4)
C7—Fe1—C10—C11	36.9 (4)	C20—C21—C22—C18	−0.5 (6)
C6—Fe1—C10—C11	−92.3 (4)	Fe2—C21—C22—C18	−59.6 (4)
C4—Fe1—C10—C11	−176.7 (4)	C20—C21—C22—Fe2	59.1 (4)
C5—Fe1—C10—C11	−136.3 (4)	C13—Fe2—C22—C18	−77.4 (4)
C8—C7—C11—C10	0.4 (7)	C16—Fe2—C22—C18	−162.1 (4)
Fe1—C7—C11—C10	60.1 (4)	C20—Fe2—C22—C18	79.9 (4)
C8—C7—C11—Fe1	−59.7 (5)	C19—Fe2—C22—C18	36.3 (4)
C9—C10—C11—C7	−1.2 (7)	C14—Fe2—C22—C18	−46.0 (9)
Fe1—C10—C11—C7	−59.7 (5)	C17—Fe2—C22—C18	−120.1 (4)
C9—C10—C11—Fe1	58.5 (4)	C15—Fe2—C22—C18	165.9 (6)
C2—Fe1—C11—C7	−89.2 (4)	C21—Fe2—C22—C18	118.3 (5)
C3—Fe1—C11—C7	−50.5 (6)	C13—Fe2—C22—C21	164.3 (3)
C8—Fe1—C11—C7	38.1 (4)	C16—Fe2—C22—C21	79.6 (4)
C9—Fe1—C11—C7	83.1 (4)	C20—Fe2—C22—C21	−38.4 (3)
C6—Fe1—C11—C7	−133.8 (4)	C18—Fe2—C22—C21	−118.3 (5)
C4—Fe1—C11—C7	142 (2)	C19—Fe2—C22—C21	−82.0 (4)
C5—Fe1—C11—C7	−173.3 (4)	C14—Fe2—C22—C21	−164.3 (7)
C10—Fe1—C11—C7	120.2 (5)	C17—Fe2—C22—C21	121.6 (4)
C2—Fe1—C11—C10	150.7 (4)	C15—Fe2—C22—C21	47.6 (8)
C3—Fe1—C11—C10	−170.7 (4)	O1—Cu1—O5—C23	28.6 (4)
C8—Fe1—C11—C10	−82.0 (4)	O4^i^—Cu1—O5—C23	119.2 (4)
C9—Fe1—C11—C10	−37.0 (4)	O2^i^—Cu1—O5—C23	−149.5 (4)
C7—Fe1—C11—C10	−120.2 (5)	O3—Cu1—O5—C23	−61.2 (4)

Symmetry codes: (i) −*x*, −*y*+1, −*z*+1.

### Hydrogen-bond geometry (Å, °)

**Table:**

*D*—H···*A*	*D*—H	H···*A*	*D*···*A*	*D*—H···*A*
O5—H5O···O6^ii^	0.82 (6)	1.95 (6)	2.759 (6)	170 (7)
O6—H6O···O2^iii^	0.84 (4)	2.18 (5)	2.925 (6)	147 (7)

Symmetry codes: (ii) −*x*+1, −*y*+1, −*z*+1; (iii) −*x*+1, −*y*, −*z*+1.
